# Investigation of Factors Affecting Early Quality of Life of Patients after Breast Cancer Surgery

**DOI:** 10.3390/healthcare9020213

**Published:** 2021-02-16

**Authors:** Yoshiteru Akezaki, Eiji Nakata, Masato Kikuuchi, Ritsuko Tominaga, Hideaki Kurokawa, Masaki Okamoto, Makiko Hamada, Kenjiro Aogi, Shozo Ohsumi, Shinsuke Sugihara

**Affiliations:** 1 Division of Physical Therapy, Kochi Professional University of Rehabilitation, Kochi 781-1102, Japan; akezakiteru@yahoo.co.jp; 2 Department of Orthopaedic Surgery, Okayama University Hospital, Okayama 700-8558, Japan; 3 Department of Rehabilitation Medicine, National Hospital Organization Shikoku Cancer Center, Ehime 791-0280, Japan; kikuuchi.masato.tu@mail.hosp.go.jp (M.K.); tominaga.ritsuko.fk@mail.hosp.go.jp (R.T.); kurokawa.hideaki.ac@mail.hosp.go.jp (H.K.); okamoto.masaki.cb@mail.hosp.go.jp (M.O.); hamada.makiko.wu@mail.hosp.go.jp (M.H.); sugihara.shinsuke.rk@mail.hosp.go.jp (S.S.); 4 Department of Breast Oncology, National Hospital Organization Shikoku Cancer Center, Ehime 791-0280, Japan; aogi.kenjiro.zx@mail.hosp.go.jp (K.A.); osumi.shozo.ur@mail.hosp.go.jp (S.O.)

**Keywords:** quality of life, breast cancer, neoadjuvant chemotherapy, postoperative chemotherapy, postoperative radiotherapy

## Abstract

Objective: The purpose of this study was to investigate factors related to early quality of life (QOL) three months after surgery in breast cancer patients with axillary lymph node dissection. Methods: The subjects of this study were 195 consecutive patients who underwent axillary lymph node dissection for breast cancer. Age, body mass index, level of lymph node dissection, marriage, children, co-resident household members, neoadjuvant chemotherapy, postoperative chemotherapy, postoperative hormonal therapy, postoperative radiotherapy, upper limb function (disabilities of the arm, shoulder, and hand (DASH)), and QOL (European Organization for the Treatment and Research of Cancer Quality of Life Questionnaire (EORTC QLQ-C30)) were evaluated. For each item of the EORTC QLQ-C30, compared with preoperative status and three months after surgery, those who improved or remained unchanged in the three months after surgery were classified as the maintenance and improved groups, and those with worsening status were classified as the worsened group. Results: Age, level of lymph node dissection, DASH, neoadjuvant chemotherapy, postoperative chemotherapy, and postoperative radiotherapy were significantly associated with QOL (*p* < 0.05). Conclusions: The early QOL of postoperative patients with breast cancer is affected by multiple factors, such as upper limb function and postoperative chemotherapy, and thus comprehensive intervention is required.

## 1. Introduction

Breast cancer is the most frequently diagnosed cancer and the most common cause of cancer-related death in females worldwide [1]. The number of breast cancer patients continues to increase. However, in Japan, 5- and 10-year relative survival was improved by 2.8% and 2.4% respectively, from 1993 to 2006 in patients with breast cancer due to advances in cancer treatment [2]. Advances in diagnosis and treatment have reduced mortality and improved survival rates in breast cancer patients. Patient-centred approaches are being emphasised in healthcare systems, and patient quality of life (QOL) is important.

The QOL of cancer patients is a subjective assessment of physical, psychological, social, and spiritual well-being factors [3,4]. In QOL evaluation of cancer patients, the Medical Outcomes Study 36-Item Short-Form Health Survey [5], widely used for chronic diseases in general, and the Functional Assessment of Cancer Therapy [6] or European Organization for the Treatment and Research of Cancer QOL Questionnaire (EORTC QLQ-C30) [7] may be used specifically for cancer. QOL is also an important indicator because it is useful for improving function, predicting mortality and survival, and determining improvements in treatment outcomes [8,9,10,11,12,13]. Cancer survivors have worse QOL compared with non-cancerous controls [14,15]. Determining the factors that affect the QOL of breast cancer patients is important in providing management, care, and rehabilitation.

In patients with breast cancer, surgery and treatments such as chemotherapy and radiotherapy (RT) may lead to pain, reduced range of motion, lymphedema, cording, stiffness, decreased strength, reduced upper limb function, and decreased activity tolerance [16,17,18,19]. Arm/shoulder problems and QOL have been reported to be associated with breast cancer [20]. Several studies have reported that breast cancer patients have a reduced QOL due to side effects from chemotherapy and radiation therapy [21,22,23,24]. Also, family situations such as divorced and unmarried women have been found to affect QOL [25]. However, in Japan, few studies have examined factors affecting QOL in patients after breast cancer surgery.

In this study, we investigated factors related to early QOL three months after surgery in breast cancer patients with axillary lymph node dissection.

## 2. Methods

### 2.1. Study Design

This was a retrospective, observational investigation of the changes in QOL following breast cancer treatment.

### 2.2. Patients and Methods

The patients of this study were 195 consecutive postoperative breast cancer patients who underwent axillary lymph node dissection at our hospital between November 2013 and December 2016. Patients who could not undergo measurement of QOL before and three months after surgery were excluded. All subjects were female. 

In this study, age, body mass index (BMI), resection of pectoralis minor muscle (yes or no), level of lymph node dissection (level 1 or level 2 and higher), marriage (yes or no), children (yes or no), co-resident household members (yes or no), neoadjuvant chemotherapy (yes or no), postoperative chemotherapy (yes or no), postoperative hormonal therapy (yes or no), postoperative radiotherapy (yes or no), upper limb function (disabilities of the arm, shoulder and hand (DASH)), and QOL were evaluated.

### 2.3. Rehabilitation Program 

In preoperative rehabilitation during hospitalisation, the patient performed upper limb exercises according to a digital versatile disc (DVD) and checked the necessary rehabilitation after surgery. In postoperative rehabilitation, exercise of distal to the elbow joint started from the first and second postoperative days. From the third postoperative day to the day of drain withdrawal, exercise and activities of daily living (ADL) of upper limbs were performed within 90 degrees of shoulder flexion and abduction. After removal of the drain, exercise and ADL of upper limbs were adjusted according to the patient’s degree of pain, with no angle limit set for the shoulder range of motion. During the hospital admission period and at discharge, a physiotherapist, an occupational therapist, or nurses provided guidance to patients on how to deal with postoperative adverse events such as axillary web syndrome and lymphedema. At the first, second, and third months after surgery, a physiotherapist and an occupational therapist evaluated the exercise implementation status of the patients and assessed their upper limb function and adverse events status. The physiotherapist and occupational therapist provided exercise, ADL guidance, and response to adverse events depending on the patient’s condition.

### 2.4. Disabilities of the Arm, Shoulder, and Hand 

DASH is a standardised upper-extremity outcome measure in the form of a self-administered questionnaire that contains 30 items on a disability/symptom scale concerning the patient’s health status [26]. The score range of DASH is from 0 to 100. Scores of 100 indicate the most severe disability of the upper extremities.

The evaluation of DASH measurements used values at three months after surgery.

### 2.5. Measurement of Quality of Life

QOL was measured using the EORTC QLQ-C30. The EORTC QLQ-30 is organised into five functional scales (physical functioning, role functioning, emotional functioning, cognitive functioning, and social functioning), eight symptom items (fatigue, nausea and vomiting, pain, dyspnoea, insomnia, appetite loss, constipation, and diarrhoea), a global health status, and perceived financial difficulties [7]. For each item, compared with the preoperative status and that three months after surgery, those who improved or remained unchanged in the three months after surgery were classified as the maintenance and improved groups, and those with worsening status were classified as the worsened group.

### 2.6. Statistical Analysis

Univariate analysis was performed using Student’s *t*-test, chi-squared test, and the Mann–Whitney U test to identify the factors associated with QOL at three months after surgery. Next, including items with *p* < 0.2 on univariate analysis, logistic regression analysis was used to identify factors that strongly influence QOL at three months after surgery. All analyses were conducted using SPSS software version 22.0 (IBM, Tokyo, Japan). All tests were two-sided and *p* < 0.05 was considered significant.

## 3. Results

### 3.1. Time Course of QOL

The time course of QOL from the preoperative days through to postoperative three months are shown in Table 1. Global health status and physical, role, emotional, and social functional scales were observed to gradually improve during the three months after surgery. Among the symptom scales, fatigue, pain, insomnia, constipation, diarrhoea, and financial difficulties were observed to gradually improve during the three months after surgery. Conversely, nausea and vomiting, dyspnoea, and appetite loss were observed to gradually worsen during the three months after surgery.

### 3.2. Univariate Analysis 

The data of the univariate analysis is not shown in Table. For global health status, neoadjuvant chemotherapy, postoperative chemotherapy, postoperative radiotherapy, and DASH were different between the two groups (*p* < 0.2). Regarding financial difficulties, age, BMI, neoadjuvant chemotherapy, postoperative chemotherapy, postoperative radiotherapy, and DASH were different between the two groups (*p* < 0.2). For physical functioning, age, level of lymph node dissection, neoadjuvant chemotherapy, postoperative chemotherapy, postoperative radiotherapy, and DASH were different between the two groups (*p* < 0.2). For role functioning, BMI, neoadjuvant chemotherapy, postoperative chemotherapy, postoperative radiotherapy, and DASH were different between the two groups (*p* < 0.2). Concerning emotional functioning, co-resident household members, postoperative radiotherapy, and DASH were different between the two groups (*p* < 0.2). Regarding cognitive functioning, children and DASH were different between the two groups (*p* < 0.2). For social functioning, neoadjuvant chemotherapy, postoperative chemotherapy, postoperative radiotherapy, and DASH were different between the two groups (*p* < 0.2). Regarding fatigue, level of lymph node dissection, neoadjuvant chemotherapy, postoperative chemotherapy, postoperative radiotherapy, and DASH were different between the two groups (*p* < 0.2). With respect to nausea and vomiting, neoadjuvant chemotherapy, postoperative chemotherapy, postoperative hormonal therapy, postoperative radiotherapy, and DASH were different between the two groups (*p* < 0.2). Regarding pain, age, neoadjuvant chemotherapy, and DASH were different between the two groups (*p* < 0.2). For dyspnoea, neoadjuvant chemotherapy, postoperative chemotherapy, postoperative hormonal therapy, postoperative radiotherapy, and DASH were different between the two groups (*p* < 0.2). For insomnia, level of lymph node dissection, neoadjuvant chemotherapy, postoperative radiotherapy, and DASH were different between the two groups (*p* < 0.2). Regarding appetite loss, neoadjuvant chemotherapy, postoperative chemotherapy, postoperative hormonal therapy, postoperative radiotherapy, and DASH were different between the two groups (*p* < 0.2). With respect to constipation, neoadjuvant chemotherapy, postoperative chemotherapy, postoperative hormonal therapy, postoperative radiotherapy, and DASH were different between the two groups (*p* < 0.2). For diarrhoea, level of lymph node dissection, postoperative chemotherapy, postoperative radiotherapy, and DASH were different between the two groups (*p* < 0.2). 

### 3.3. Logistic Regression Analysis

The results of the logistic regression analysis are shown in Table 2 and Table 3. In global health status, recovery was worse with high DASH (*p* < 0.05). In physical functioning, the recovery was worse with no neoadjuvant chemotherapy and high DASH (*p* < 0.05). In role functioning, the recovery was worse with no neoadjuvant chemotherapy and high DASH (*p* < 0.05). In emotional functioning, the recovery was worse with the no radiotherapy and high DASH (*p* < 0.05). In cognitive functioning, the recovery was worse with high DASH (*p* < 0.05). In social functioning, the recovery was worse with high DASH (*p* < 0.05). For fatigue, recovery was worse with the no neoadjuvant chemotherapy and high DASH (*p* < 0.05). For nausea and vomiting, recovery was worse with the no postoperative chemotherapy and high DASH (*p* < 0.05). For pain, recovery was worse with older age, no neoadjuvant chemotherapy, and high DASH (*p* < 0.05). For dyspnoea, recovery was worse with no neoadjuvant chemotherapy and high DASH (*p* < 0.05). For insomnia, the recovery was worse with high DASH (*p* < 0.05). In appetite loss, recovery was worse with no neoadjuvant chemotherapy and high DASH (*p* < 0.05). For constipation, recovery was worse with high DASH (*p* < 0.05). For diarrhoea, recovery was worse with lymph node dissection level 2 or more, no postoperative chemotherapy, and high DASH (*p* < 0.05).

## 4. Discussion

In patients undergoing breast cancer surgery, QLQ-C30 changes up to three months after surgery showed that the physical, role, emotional, and social functions of the overall condition and functional scales were relatively improved. However, on the symptom scale, nausea and vomiting, dyspnoea, and loss of appetite tended to worsen by three months after surgery.

Arm/shoulder problems and QOL have been reported in breast cancer patients [20]. Furthermore, arm/shoulder problems were associated with reductions in mental and physical QOL eight years after the diagnosis of breast cancer [27]. In this study, we examined factors affecting QOL improvement in the three months after breast cancer surgery. DASH affected the improvement of all items of the global health scale and functional scale of QOL. The global health scale of patients with breast cancer surgery may be strongly influenced by improved upper limb function. On the functional scale, physical functioning and role functioning are movements using the upper limbs, and thus DASH may have been influenced. Although emotional functioning, cognitive functioning, and social functioning are not aspects of QOL directly related to upper limb function, secondary effects were considered due to the reduction in upper limb function. As for the symptom items of QLQ-C30, DASH affected all items. DASH showed that patients with upper limb function symptoms had a poor improvement in QLQ-C30 symptom items because DASH included items of upper limb function symptoms. Therefore, rehabilitation intervention to improve upper limb function is important because early QOL of patients after breast cancer surgery is strongly affected by upper limb dysfunction.

Several studies have shown that chemotherapy significantly reduces the QOL of patients with breast cancer [21,22]. Breast cancer patients treated with chemotherapy experienced a significantly negative impact of adjuvant chemotherapy on QOL compared with patients treated with radiation therapy and adjuvant hormone therapy [28]. This study identified that the presence or absence of chemotherapy affected nausea and diarrhoea. After chemotherapy, decreased QOL is associated with anorexia, anaemia, nausea, and vomiting [29,30]. In this study, drug-induced side effects were not examined because the different chemotherapy types were not included in the survey items. However, side effects of chemotherapy may affect improvements in nausea and diarrhoea in QLQ-C30.

In this study, the presence or absence of NAC affected improvements in physical functioning, role functioning, and the symptoms of fatigue, pain, dyspnoea, and appetite loss. Although NAC reduced physical functioning, role functioning, fatigue, pain, dyspnoea, and appetite loss due to side effects before surgery, they had improved three months after surgery, and hence patients who underwent NAC may have improved.

Radiation therapy had a positive effect on emotional functioning in patients. Patients who have not undergone radiation therapy include those who have undergone chemotherapy and hormonal therapy and those who have had no adjuvant therapy. Patients who receive chemotherapy and hormonal therapy have psychological effects due to side effects, and patients without adjuvant therapy have psychological burdens due to the lack of treatment options. Therefore, the presence or absence of radiation therapy affect emotional functioning.

Pain, fatigue, insomnia, and mood disturbance are highly prevalent in elderly patients who have undergone cancer therapy [31]. In this study, the recovery of pain in QLQ-C30 showed a lower degree of improvement for older patients. Older patients may feel less useful in life and may be affected by psychological aspects such as uncertainty about the future [32]. Although the decline in resilience due to aging may be a cause of pain persistence after surgery and adjuvant therapy, the results of this study alone are unclear.

Divorced status or the presence of no partner have been shown to be risk factors for poor QOL in women with breast cancer [25,29]. Also, married or cohabiting patients had better QOL scores than patients living alone or who were divorced [33,34]. In the present study, QOL was not associated with marriage or unmarried status, children, and co-resident household members. However, financial and emotional support from partners is important in enhancing QOL [25]. Although marriage or unmarried status, children, and co-resident household members were not significant factors in this study, we have not investigated the impact of family support. Therefore, in this study, the relationship between family status and QOL is not clear.

Although our study is not an intervention study compared with a non-rehabilitation control group, we have determined factors that affect QOL of breast cancer patients. Thus, it is suggested that medical staff need to understand relevant factors that affect QOL in breast cancer patients and plan and provide management, care, and rehabilitation for them.

### Study Limitations

There were limitations in the present study. This study investigated QOL at three months, but the results may differ when long-term QOL is investigated. In addition, this study was a single-centre study and multicentre research was not conducted, therefore there may be differences between facilities.

## 5. Conclusions

Factors affecting QOL three months after surgery were examined in breast cancer patients with axillary lymph node dissection. The early QOL of postoperative patients with breast cancer is affected by multiple factors such as upper limb function and postoperative chemotherapy, and thus comprehensive intervention is required.

## Figures and Tables

**Table 1:** The time course of quality of life (QOL) from preoperative to three months. Mean ± standard deviation.

	Before Surgery	One Week	One Month	Two Months	Three Months
Global health status/QOL	62.9 ± 23.0	56.9 ± 23.1	64.2 ± 20.7	64.8 ± 21.7	65.4 ± 24.1
Physical function	89.6 ± 15.0	78.5 ± 19.2	85.8 ± 11.6	86.3 ± 13.8	87.6 ± 12.0
Role function	87.8 ± 21.1	57.9 ± 30.4	75.8 ± 18.3	77.8 ± 23.3	81.5 ± 22.1
Emotional function	74.3 ± 19.8	76.2 ± 19.1	80.7 ± 17.5	83.2 ± 16.6	85.8 ± 15.6
Cognitive function	83.8 ± 19.1	85.1 ± 17.4	86.2 ± 15.7	84.3 ± 17.6	85.1 ± 17.9
Social function	82.2 ± 21.7	69.7 ± 31.1	80.6 ± 21.7	79.2 ± 21.8	83.9 ± 22.0
Fatigue	23.4 ± 21.1	28.7 ± 18.8	26.9 ± 19.0	26.4 ± 19.3	25.9 ± 18.4
Nausea/Vomiting	1.5 ± 6.5	3.2 ± 8.5	1.7 ± 7.0	4.3 ± 11.5	4.0 ± 12.4
Pain	15.1 ± 19.2	35.2 ± 22.4	28.9 ± 16.3	20.2 ± 17.4	18.8 ± 19.9
Dyspnoea	11.6 ± 19.5	8.3 ± 16.0	9.9 ± 16.4	11.7 ± 18.8	12.5 ± 18.8
Insomnia/sleep	18.1 ± 23.3	34.6 ± 26.7	20.8 ± 22.8	22.7 ± 26.5	20.2 ± 22.8
Appetite loss	11.1 ± 20.2	9.3 ± 18.2	7.5 ± 16.6	13.2 ± 21.9	12.3 ± 20.2
Constipation	16.2 ± 22.3	17.5 ± 31.8	11.2 ± 17.1	17.7 ± 22.4	16.2 ± 23.5
Diarrhoea	7.3 ± 16.1	7.4 ± 14.3	4.3 ± 13.2	8.8 ± 18.8	6.3 ± 16.2
Financial hardship related to illness	21.7 ± 24.9	27.5 ± 31.8	18.3 ± 23.6	19.5 ± 23.4	15.0 ± 21.2

**Table 2:** Factors predictive of physical functioning, role functioning, emotional functioning, cognitive functioning, and social functioning of European Organization for the Treatment and Research of Cancer Quality of Life Questionnaire: logistic regression analyses.

	Variable	Odds Ratio (95% CI)	*p*-Value
Global health status/QOL	Neoadjuvant chemotherapy	2.443 (0.966–6.181)	0.059
	Postoperative chemotherapy	1.306 (0.573–2.975)	0.525
	Postoperative radiotherapy	2.395 (0.927–6.182)	0.071
	DASH	0.938 (0.912–0.965)	*p* < 0.0001
Physical functioning	Age	1.031 (0.999–1.063)	0.057
	Level of lymph node dissection	1.550 (0.660–3.639)	0.314
	Neoadjuvant chemotherapy	8.870 (3.265–24.098)	*p* < 0.0001
	Postoperative chemotherapy	1.473 (0.612–3.545)	0.388
	Postoperative radiotherapy	1.493 (0.593–3.757)	0.395
	DASH	0.951 (0.925–0.979)	0.001
Role functioning	Body mass index	1.079 (0.983–1.185)	0.108
	Neoadjuvant chemotherapy	2.766 (1.058–7.236)	0.038
	Postoperative chemotherapy	0.802 (0.341–1.885)	0.613
	Postoperative radiotherapy	1.576 (0.595–4.175)	0.360
	DASH	0.928 (0.899–0.957)	*p* < 0.0001
Emotional functioning	Co-resident household members	2.781 (0.856–9.038)	0.089
	Postoperative radiotherapy	3.984 (1.406–11.288)	0.009
	DASH	0.966 (0.939–0.994)	0.019
Cognitive functioning	Children	0.527 (0.201–1.382)	0.193
	DASH	0.961 (0.936–0.985)	0.002
Social functioning	Neoadjuvant chemotherapy	2.128 (0.679–6.677)	0.195
	Postoperative chemotherapy	0.732 (0.278–1.929)	0.528
	Postoperative radiotherapy	1.996 (0.621–6.413)	0.246
	DASH	0.947 (0.921–0.974)	*p* < 0.0001

CI: confidence interval, QOL: quality of life, DASH: Disabilities of the Arm, Shoulder, and Hand.

**Table 3:** Factors predicting of fatigue, nausea and vomiting, pain, and dyspnoea of European Organization for the Treatment and Research of Cancer Quality of Life Questionnaire: logistic regression analyses.

	Variable	Odds Ratio (95% CI)	*p*-Value
Fatigue	Level of lymph node dissection	1.780 (0.781–4.054)	0.170
	Neoadjuvant chemotherapy	3.736 (1.522–9.168)	0.004
	Postoperative chemotherapy	0.999 (0.450–2.217)	0.999
	Postoperative radiotherapy	1.890 (0.768–4.650)	0.166
	DASH	0.968 (0.943–0.992)	0.011
Nausea and vomiting	Neoadjuvant chemotherapy	4.927 (0.848–28.636)	0.076
	Postoperative chemotherapy	0.127 (0.020–0.791)	0.027
	Postoperative hormonal therapy	4.858 (0.558–42.335)	0.152
	Postoperative radiotherapy	0.342 (0.062–1.894)	0.219
	DASH	0.967 (0.938–0.997)	0.033
Pain	Age	0.972 (0.945–1.000)	0.046
	Neoadjuvant chemotherapy	2.314 (1.144–4.682)	0.020
	DASH	0.953 (0.928–0.978)	*p* < 0.0001
Dyspnoea	Neoadjuvant chemotherapy	5.321 (1.027–27.565)	0.046
	Postoperative chemotherapy	0.651 (0.163–2.606)	0.544
	Postoperative hormonal therapy	1.415 (0.355–5.644)	0.623
	Postoperative radiotherapy	2.512 (0.590–10.701)	0.213
	DASH	0.959 (0.932–0.987)	0.005
Insomnia	Level of lymph node dissection	2.059 (0.789–5.376)	0.140
	Neoadjuvant chemotherapy	2.076 (0.789–5.463)	0.139
	Postoperative radiotherapy	1.035 (0.438–2.442)	0.938
	DASH	0.962 (0.938–0.987)	0.003
Appetite loss	Neoadjuvant chemotherapy	3.841 (1.045–14.120)	0.043
	Postoperative chemotherapy	0.480 (0.137–1.683)	0.251
	Postoperative hormonal therapy	1.448 (0.422–4.973)	0.556
	Postoperative radiotherapy	0.900 (0.257–3.158)	0.869
	DASH	0.959 (0.933–0.986)	0.003
Constipation	Neoadjuvant chemotherapy	2.589 (0.774–8.658)	0.122
	Postoperative chemotherapy	0.644 (0.194–2.143)	0.473
	Postoperative hormonal therapy	1.612 (0.488–5.323)	0.434
	Postoperative radiotherapy	1.195 (0.354–4.036)	0.775
	DASH	0.972 (0.947–0.998)	0.038
Diarrhea	Level of lymph node dissection	0.278 (0.090–0.858)	0.026
	Postoperative chemotherapy	0.165 (0.031–0.874)	0.034
	Postoperative radiotherapy	1.194 (0.239–5.961)	0.829
	DASH	0.960 (0.929–0.991)	0.013
Financial difficulties	Age	1.042 (0.987–1.100)	0.140
	Body mass index	1.079 (0.903–1.290)	0.403
	Neoadjuvant chemotherapy	6.441 (0.636–65.237)	0.115
	Postoperative chemotherapy	1.287 (0.277–5.982)	0.747
	Postoperative radiotherapy	2.395 (0.352–16.315)	0.372
	DASH	0.971 (0.937–1.007)	0.112

CI: confidence interval, DASH: Disabilities of the Arm, Shoulder and Hand.

## Data Availability

The data presented in this study are available on request from the corresponding author. The data are not publicly available due to Participant’s personal information.
